# Seeding and Growth of β-Amyloid Aggregates upon Interaction with Neuronal Cell Membranes

**DOI:** 10.3390/ijms21145035

**Published:** 2020-07-16

**Authors:** Álvaro Ruiz-Arias, Jose M. Paredes, Chiara Di Biase, Juan M. Cuerva, María D. Giron, Rafael Salto, Juan A. González-Vera, Angel Orte

**Affiliations:** 1 Departamento de Fisicoquímica, Unidad de Excelencia de Química Aplicada a Biomedicina y Medioambiente, Facultad de Farmacia, Universidad de Granada, Campus Cartuja, 18071 Granada, Spain; alvaroruizarias@ugr.es (Á.R.-A.); jmparedes@ugr.es (J.M.P.); c.dibiase@campus.uniurb.it (C.D.B.); gonzalezvera@ugr.es (J.A.G.-V.); 2 Department of Biomolecular Sciences, University of Urbino Carlo Bo, 61029 Urbino, Italy; 3 Departamento de Química Orgánica, Unidad de Excelencia de Química Aplicada a Biomedicina y Medioambiente, Facultad de Ciencias, Universidad de Granada, Campus Fuentenueva, 18071 Granada, Spain; jmcuerva@ugr.es; 4 Departamento de Bioquímica y Biología Molecular II, Unidad de Excelencia de Química Aplicada a Biomedicina y Medioambiente, Facultad de Farmacia, Universidad de Granada, Campus Cartuja, 18071 Granada, Spain; mgiron@ugr.es (M.D.G.); rsalto@ugr.es (R.S.)

**Keywords:** neurodegenerative disease, amyloid, misfolding, Alzheimer’s disease, fluorescence microscopy, FRET

## Abstract

In recent years, the prevalence of amyloid neurodegenerative diseases such as Alzheimer’s disease (AD) has significantly increased in developed countries due to increased life expectancy. This amyloid disease is characterized by the presence of accumulations and deposits of β-amyloid peptide (Aβ) in neuronal tissue, leading to the formation of oligomers, fibers, and plaques. First, oligomeric intermediates that arise during the aggregation process are currently thought to be primarily responsible for cytotoxicity in cells. This work aims to provide further insights into the mechanisms of cytotoxicity by studying the interaction of Aβ aggregates with Neuro-2a (N2a) neuronal cells and the effects caused by this interaction. For this purpose, we have exploited the advantages of advanced, multidimensional fluorescence microscopy techniques to determine whether different types of Aβ are involved in higher rates of cellular toxicity, and we measured the cellular stress caused by such aggregates by using a fluorogenic intracellular biothiol sensor. Stress provoked by the peptide is evident by N2a cells generating high levels of biothiols as a defense mechanism. In our study, we demonstrate that Aβ aggregates act as seeds for aggregate growth upon interacting with the cellular membrane, which results in cell permeability and damage and induces lysis. In parallel, these damaged cells undergo a significant increase in intracellular biothiol levels.

## 1. Introduction

In recent years, neurodegenerative diseases have become a major threat to public health in industrialized countries, since the prevalence of these disorders increases as the percentage of elderly people in the population rises. Symptoms of neurodegenerative diseases begin many years after the onset of illness, making it extremely difficult to provide an effective and early diagnosis. The common feature of amyloid diseases is the aberrant aggregation of a certain protein, initially in small oligomers, which subsequently grow to form various fibrillar structures and plaques. These illnesses promote the progressive displacement of neurons along with secondary alterations due to effects on the central nervous system [1]. Approximately forty neurodegenerative diseases, including Alzheimer’s disease (AD), are characterized by the presence of insoluble amyloid deposits in the affected tissues [2]. A hallmark of AD is the existence of plaques formed by β-amyloid (Aβ) fibers in the neocortex and hippocampus of the brain [3,4]. When the transmembrane amyloid precursor protein is proteolyzed by β- and γ-secretase, it is cleaved into small peptide fragments, namely, (Aβ) peptides [5,6,7]. The initial oligomeric structures that emerged during the Aβ aggregation process are thought to be the main sources of cytotoxicity related to AD, and they are currently one of most interesting pharmacological targets, as these oligomeric structures seem to be essential in the neurotoxicity of amyloid structures [8]. Among all Aβ isoforms, the Aβ (1–42) peptide (Aβ42) seems to be the most neurotoxic species compared to the other common 40-residue fragment. The different behaviors of these two peptides are striking, especially due to the fact that only two extra C-terminal residues are present in the Aβ42 form [9,10].

The interaction at a cellular level of such amyloid oligomers is an important step towards disease progression. During amyloidogenic misfolding, specific proteins undergo aberrant three-dimensional self-interactions. These structures expose solvent hydrophobic regions that are usually hidden in the native state. This fact facilitates protein aggregation and the subsequent nucleation into β-sheet-rich structures [11]. Thus, this process entails not only the loss of the physiological function but also the generation of abnormal protein aggregates. These filamentary oligomers rich in β-sheet structures exhibit unstable behavior and can alter the chemical composition of the extracellular matrix [12]. Recent studies have suggested that oligomeric intermediates formed throughout amyloidogenic aggregation are more toxic than mature fibrils. For instance, a slow conversion of early, easy-degradable aggregates into compact, highly structured oligomers was detected in α-synuclein fibrillization [13], which is related to the progress of Parkinson’s disease. These later oligomers induce much higher levels of reactive oxygen species (ROS) in cells than do earlier oligomers [14]. While focusing on Aβ aggregation, these oligomers interact with lipid membrane surfaces through weak electrostatic interactions that promote conformational transition from α-helix structures to β-sheet conformations [15]. Moreover, it has been observed that Aβ alters membrane phospholipid fluidity through its own insertion into lipid bilayers [16]. That insertion, in form of pores, induces calcium-signaling, activating NADPH oxidase, stimulating nitric oxide production, and finally leading to increased oxidative stress and to neurodegeneration [14,17,18,19].

The urgent need for reliable tools that provide further information on the cellular effect of amyloid aggregates make this a very active field of research. For instance, the use of fluorescent probes to study relevant intracellular processes such as the interaction of the peptide with neuronal cells is highly advantageous due to its low invasiveness [20,21]. One of the most important steps to understand the aberrant protein aggregation process is the identification and characterization of different species formed during the misfolding process. A widely used technique to characterize amyloid fibers has been the use of thioflavin T (ThT) as a fluorophore, as its interaction with amyloid fibers enhances fluorescence. However, its binding to early aggregates is weak, so this dye cannot be used for the characterization of early oligomers [22]. Thus, new probes, such as specific labeling antibodies [23] and especially fluorescent reporters, are continually being developed for the specific and sensitive detection of the early species of aggregation, as well as advanced techniques that allow the visualization and characterization of such aggregates [22,24,25]. For instance, we have recently described a new family of highly solvatochromic and fluorogenic dyes based on the quinolimide scaffold [26], named 9-amino-quinolimides, and these dyes have shown great potential for probing β-amyloid aggregation in a zebrafish model, identifying the formation of different types of early oligomers [27]. Due to its noninvasive nature and high sensitivity, this technique allows intracellular processes to be studied. Among confocal fluorescence microscopy techniques, fluorescence lifetime imaging microscopy (FLIM) has been indicated to be an invaluable tool to characterize amyloid aggregates [28,29,30], provided its multidimensional capabilities, in terms of emission intensity, fluorescence lifetime, τ, and intraoligomer fluorescence resonance energy transfer (FRET) efficiency, in particular through a powerful microscopy variant called dual-color pulsed interleaved excitation FLIM (PIE-FLIM).

Once specific tools to decipher different types of amyloidogenic aggregates are in place, it is also important to be capable of studying the direct mechanisms involved in cellular toxicity. For instance, in a previous work, an ultracentrifugation gradient method has been described to separate between different types of Aβ aggregates according to their size and density [31]. Using this methodology, in another work, the toxicity of the corresponding aggregates of Aβ42 was monitored by using two methods capable of measuring the cytotoxic effect of aggregates: a quantitative assay to determine the ability of peptide aggregates to disorganize the structure of lipid bilayers and an assay that measures the production of cytokines from microglial cells as an estimation of the inflammation capacity of the different aggregates. With these methods, it was demonstrated that the larger the size of the aggregates is, the lower their capacity to disorganize lipid bilayers. However, a relatively increased ability to cause inflammation was observed as the size increases. These facts are correlated with structural changes in the different aggregates with different sizes and structures [8]. The effect of the aggregates of Aβ40 and Aβ42 peptides on neuronal cells as a function of their dose has also been studied [32], showing an increase in ROS generated by neurons as the concentration of added peptide rises.

Herein, we provide additional tools to characterize and understand the cellular effect of Aβ42 amyloid aggregates. We have used the Aβ42 peptide in our assays since it is believed to be one of the most important alloforms due to the significant differences in toxicity, aggregation mechanism, and physiological function that the Aβ42 peptide has with respect to other isoforms [33,34]. Aβ42 has a greater tendency to present more prefibrillary oligomeric states than Aβ40 [35]. We have studied the interaction process of the Aβ42 peptide with Neuro-2a (N2a) neuronal cells (a fast-growing mouse neuroblastoma cell line) by PIE-FLIM to distinguish different subpopulations of aggregates interacting with cells and causing lysis. Furthermore, we have also explored the cellular toxicity in real-time and at the single-cell level by monitoring the entire interaction process of the peptide with neurons over time and by evaluating cellular stress through the generation rate of biothiols using a fluorogenic biothiol probe.

## 2. Results

### 2.1. Interaction between Aβ42 Aggregates and N2a Cells

In previous works, we used PIE-FLIM imaging to directly detect and characterize amyloidogenic aggregates at a molecular level. Using this technique, it has been possible to unravel structural changes over time in amyloid aggregates, thereby characterizing different species of oligomers [28,29,30]. For instance, we unequivocally detected three different kinds of aggregates in the early stages of fibrillization of an SH3 domain [30], a validated model employed to study amyloid aggregation.

Herein, we use this technique for the first time to visualize such aggregates interacting with single cells, thus obtaining a more complete view of the process. In particular, we have focused our attention on the interaction between Aβ42 and N2a cells to analyze the effect that early aggregates and amyloid structures induce on cells and what possible interactions may exist. PIE-FLIM provides information on intraoligomer FRET efficiency as a measure of aggregate compactness. For imaging Aβ42 aggregates using PIE-FLIM, the peptide monomers must be fluorescently labeled with either a suitable energy donor or acceptor fluorophore. We employed commercial Aβ42 peptides labeled with the HiLyte Fluor 488 or HiLyte Fluor 647 (Aβ-488 and Aβ-647, respectively) fluorophore and incubated equimolar mixtures of Aβ-488 and Aβ-647 for 15 h at a concentration of 0.5 μM in SSPE buffer (sodium chloride 150 mM, sodium phosphate 10 mM, and ethylenediaminetetraacetic acid, EDTA, 1 mM), pH 7.4, and with agitation at 260 rpm. Different aliquots of the incubating mixture were taken and added to the buffer containing the N2a cells, for a final peptide concentration of 50 nM. Cells were imaged using PIE-FLIM every 10 min to follow the evolution of the interaction of Aβ42 aggregates with cells.

**Figure 1:**
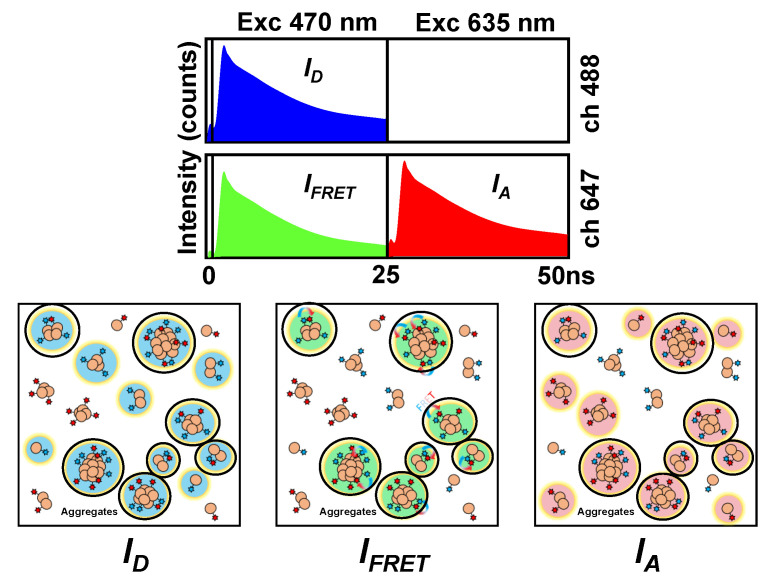
Dual-color pulsed interleaved excitation–fluorescence lifetime imaging microscopy (PIE-FLIM) scheme and aggregate selection criteria. The three different time gates distributed in the two detection channels define the reconstructed *I_D_*, *I_FRET_*, and *I_A_* images. The three images are then analyzed to identify fluorescent events. Only coincident pixels in all three images are selected as aggregates (indicated with dark circles in the cartoon).

The combination of the PIE excitation scheme, by which one of the lasers directly excites the donor fluorophore and the second laser, temporarily delayed, excites the acceptor fluorophore, with the collection of fluorescence emission in two detection channels (one for the donor and one for the acceptor) allows for specific time gates to be set to discriminate photons and reconstruct three different FLIM images, i.e., donor, FRET, and directly excited acceptor (see Figure 1 and the Section 4 for a complete description of the instrumentation). Pixels that colocalize simultaneously in the three images are those unequivocally selected as aggregates (Figure 1). The FRET efficiency and therefore the degree of compactness of the aggregates are related to the decrease in the fluorescence lifetime of the donor fluorophore, *τ_D_*.

**Figure 2:**
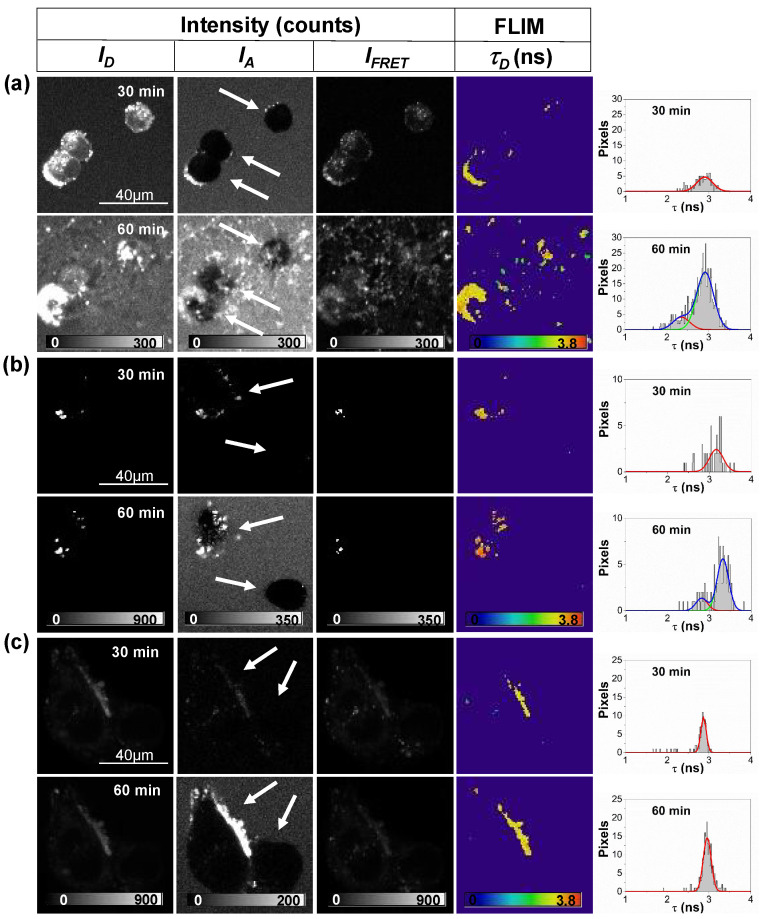
Fluorescence intensity images in the three detection windows (*I_D_*, *I_FRET_*, and *I_A_*) and FLIM images of the donor fluorophore, *τ_D_*, of equimolar mixtures of labeled Aβ-488 and Aβ-647 peptides (incubated for 15 h) interacting with Neuro-2a (N2a) cells for 30 and 60 min. Histograms in the rightmost column represent *τ_D_* distributions for only pixels selected as aggregates. The distributions were fitted to one or two (if needed) Gaussian functions (lines). Panels (**a**–**c**) are representative images from different, repeated experiments. The arrows indicate different detected cells for identification purposes.

Figure 2 shows representative examples of the strong interaction of the incubated Aβ42 peptides with undifferentiated neuronal cells. As a common feature, we found that aggregates of Aβ42 surrounded cell membranes, interacting with them after 20–30 min of incubation. After 30 min of interaction, the number of Aβ42 aggregates surrounding the cell membrane increased, even inducing cell permeability, and entering the cell cytoplasm. Usually, after 60 min of interaction, N2a cells that had aggregates on the membrane were completely lysed. This behavior is particularly noticeable in Figure 2a, in which all cells appeared to be lysed after 60 min of interaction.

Interestingly, Figure 2b shows a cell in which the aforementioned effect is clearly detected. This is the accumulation of Aβ42 aggregates on the membrane, causing total lysis at the end of the studied process. However, in this experiment, it is evident that Aβ42 aggregates concentrate on one cell and leave a second cell intact. Aggregates surround the membrane and increase the size of the initial deposits within the cell over time. Surprisingly, the second cell remains intact and free from interacting aggregates, even after 60 min. The fact that aggregates grow in size and cooperatively seem to attract more aggregates within the cellular membrane holds important implications respect to cellular toxicity, and the effect of aggregated nuclei seems to act as seeds directly on the cell membrane. Figure 2c also shows interesting results, as aggregates appeared and interacted with the cell membrane of one cell and then progressively increase the size of the deposits formed to form considerable accumulations, however, these aggregates did not attack a second, nearby cell. In this case, even the cell with aggregates stuck on the membrane maintained structural integrity. This is a very important result that was consistently found across all of our repetitions. In fact, given the low amounts of peptide used (50 nM), we frequently found cells that did not show interacting aggregates. These cells remained unharmed within the time frame of the experiments.

Importantly, the explanation for this differential behavior can be rationalized using the *τ_D_* FLIM images and the corresponding frequency histograms. As one can see in Figure 2a,b, for the lysed cells, an earlier (first) population of aggregates surrounds cell membranes, but at the end of the process, a later (second) population of aggregates, which are characterized by a lower *τ_D_* and hence are structurally more compact, is detected. The increasing emission intensity in the *I_FRET_* images and the decreased *τ_D_* values obtained over time indicate a larger FRET efficiency and an increased compactness of the aggregates, as previously shown in single-molecule FRET studies [13,30]. Strikingly, this second population was not detected in the cell that remained intact, as shown in Figure 2c. This suggests that such compact aggregates may play a crucial role in inducing cellular lysis, contributing to different toxicity mechanisms, specifically in the induction of membrane permeability and inflammation. This is in agreement with previous studies that show the high capability of small soluble aggregates to permeabilize the membrane, which is due to their high tendency to interact and cross the membrane, unlike what happens with larger aggregates [8]. Additional images showing more time points (each 10 min) for the experiments in Figure 2 can be found in Figures S1–S3 and an additional example of a repeated experiment is depicted in Figure S4 in the Supplementary Materials (SM).

### 2.2. Study of Cellular Stress Using a Biothiol Probe

In the previous experiments we confirmed how Aβ42 peptide aggregates are able to interact with neuronal cells and induce their lysis. It is thus reasonable to relate this interaction to an increase in cellular stress. Increased levels of biothiols in the cell matrix is a common physiological response as a defense mechanism associated with cellular stress [36]. Therefore, measuring the amount of intracellular biothiols generated upon interacting with pre-amyloid aggregates results in a striking way to correlate the physiological effect of cytotoxic oligomers. Generated ROS in an ensemble population of cultured cells upon addition of amyloid aggregates has commonly been reported as a measure of cellular stress and cytotoxicity [13,37,38]. However, herein, we are more interested in measuring this effect at the single-cell level.

Recently, our research group developed a fluorogenic probe to measure oxidative stress by monitoring changes in biothiol levels [38]. This probe chemically combines a highly electron-deficient 2,4-dinitrophenylsulfonyl (DNBS) group, acting as an electron sink in a photoinduced electronic transfer (PET), with a fluorescent xanthene derivative, 9-[1-(4-tert-butyl-2-methoxyphenyl)]-6-hydroxy-3H-xanthen-3-one (Granada Green, GG). The GG-DNBS probe is nonfluorescent due to PET-induced deactivation. Desulfonation of the DNBS group in the presence of thiols releases the fluorophore, resulting in an increased fluorescence emission. The GG-DNBS probe has already been successfully validated in 661W photoreceptor-derived cells [38].

**Figure 3:**
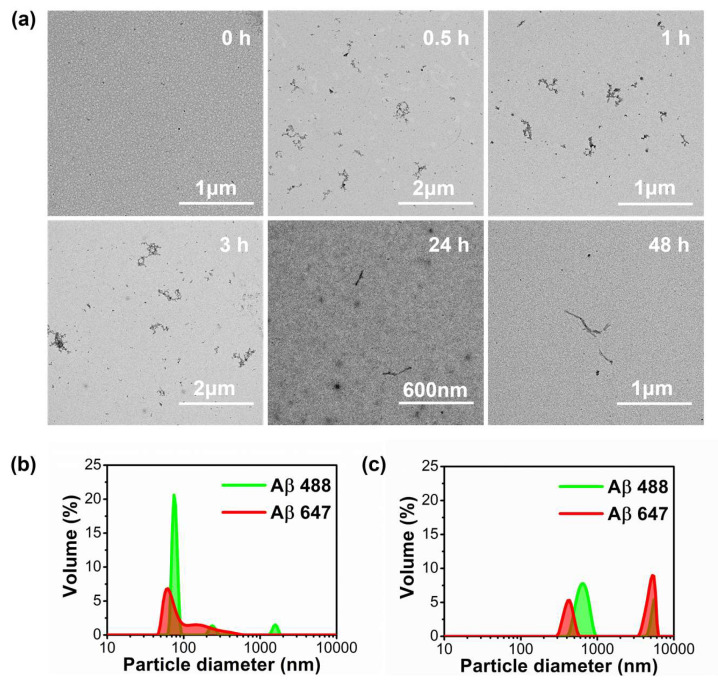
(**a**) Transmission electron microscopy (TEM) images of fluorescently labeled Aβ aggregates and fibers (equimolar 0.5 μM mixture of Aβ-488 and Aβ-647) at different incubation times. (**b**,**c**) dynamic light scattering (DLS) images of labeled Aβ peptides (Aβ-488: green line and Aβ-647: red line) formed at 0 h (**b**) and 0.3 h (**c**) of the incubation process, sonicated in a quartz cuvette in an NH_3_ solution (pH 12) to avoid their aggregation (**b**) and in a phosphate buffered saline solution pH 7.4 after 20 min of aggregation (**c**).

In order to quantify the cellular stress caused by Aβ42 aggregates interacting with N2a cells at the single-cell level, we carried out imaging experiments using the fluorogenic biothiol sensor GG-DNBS, and determined the speed at which biothiols are generated by neuronal cells when Aβ42 aggregates are added. For these experiments, we collected aliquots of the incubating samples of Aβ-647 at different time points of incubation (0, 0.5, 1, 3, 24, and 48 h) to investigate the effect of a wide range of peptide aggregation stages. The selected incubation times are important, as evidenced by transmission electron microscopy (TEM) and dynamic light scattering (DLS) measurements (Figure 3). As the incubation proceeded, curly protofibrillar structures were detected early, between 0.5 and 3 h of incubation, in TEM images, whereas longer, straight fibrils were found after 24 and 48 h of incubation (Figure 3a). The nonincubated peptides (0 h of incubation) exhibited certain degree of amorphous aggregation (Figure 3b), usually attributed to hydrophobically-driven contacts and micellar structures [39,40]. However, as shown in Figure 3c, the size of the aggregates increases considerably after 0.3 h of the aggregation process.

**Figure 4:**
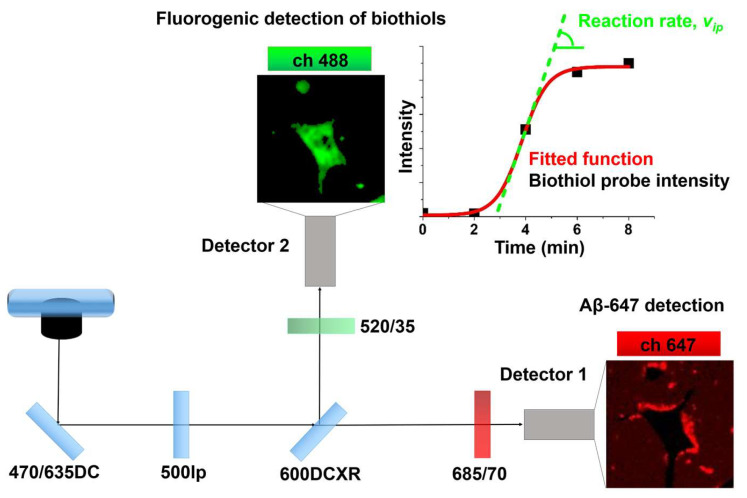
Scheme of the configuration of the dual-channel PIE-FLIM to simultaneously obtain Granada Green-2,4-dinitrophenylsulfonyl (GG-DNBS) biothiol probe and Aβ-647 aggregates images. For determining the rate of the fluorogenic biothiol reaction, the average pixel intensity in the regions of interest (black points) were fitted to a sigmoidal function (line red) and the slope at the inflection point (line green) was calculated.

Once the aliquots were collected, we added simultaneously the Aβ-647 aggregates and the GG-DNBS biothiol probe to living N2a cells. Herein, we also employed a dual-channel PIE microscopy setting; however, because the fluorescence emission of the GG-DNBS sensor is in the green spectral region, we used only the incubated red-labeled Aβ-647 peptide for the pre-amyloid aggregates. Accordingly, we used the two fluorescence intensity channels to simultaneously image the biothiol sensor response in the green channel and the Aβ-647 aggregates in the red channel (Figure 4). With this setting, we followed the kinetics of the biothiol-mediated fluorogenic reaction of GG-DNBS for 1 h by extracting the average fluorescence intensity in pixels of interest (corresponding with cell cytoplasm) as a function of time. Repetitions under same experimental conditions were carried out for each aggregation time of Aβ-647 and compared these results with those from cells in the basal state (control cells without Aβ-647 aggregates).

An important novelty of the present work, compared to previous studies using fluorogenic biothiol sensors, is the method for data analysis and the parameter of interest. In previous works with biothiol sensors, we measured the total reached intensity and/or the area under the curve of the fluorogenic reaction as the parameter related to biothiol levels [38,41]. Herein, we go a step further and focus on the actual kinetics of the fluorogenic reaction by extracting the rate of the fluorescence increase. In order to compare the effect caused by different types of aggregates and the control cells, we selected the maximum rate of the fluorogenic reaction as the parameter of interest, as this is the rate in the inflection point of the intensity (*I*) vs. time plot (see Figure 4). To do this, we obtained the plots of average *I* from GG-DNBS in the selected pixels as a function of time (*t*), and fit these plots to a sigmoidal, dose–response function:

(1) $$I=I_{0}+\frac{I_{\max}-I_{0}}{1+10^{p\left(t_{ip-t}\right)}}$$

where *I*_max_ and *I*_0_ represent the final and initial intensity values, respectively; *t_ip_* is the time of the inflection point; and *p* is related to the geometry of the sigmoidal shape.

By deriving Equation (1), we obtained the speed of the fluorogenic reaction (*v*) at each time point:

(2) $$v=\left(\frac{dI}{dt}\right)=\frac{\ln(10)\cdot \left(I_{\max}-I_{0}\right)\cdot p\cdot 10^{p\left(t_{ip}-t\right)}}{{\left[1+10^{p\left(t_{ip}-t\right)}\right]}^{2}}$$

which can be evaluated at *t* = *t_ip_* to yield the rate at the inflection point (*v_ip_*):

(3) $$v_{ip}={\left(\frac{dI}{dt}\right)}_{t_{ip}}=\frac{\ln(10)\cdot \left(I_{\max}-I_{0}\right)\cdot p}{4}$$

**Figure 5:**
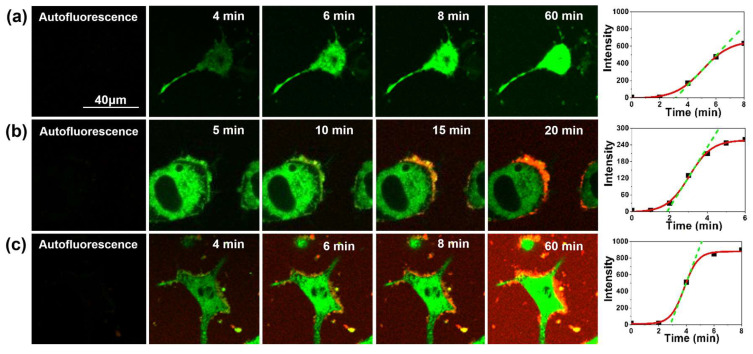
Representative images of cellular stress assays with the biothiol probe (green channel) at different times without control, (**a**) or with the addition of Aβ-647 aggregates (red channel) previously incubated for 24 h (**b**) and 48 h (**c**). The plots in the rightmost column represent the corresponding average intensity per pixel value of the biothiol probe as a function of time along with the fitted sigmoidal function (red curve) and the slope at the inflection point (green, dashed line).

The rate of the fluorogenic reaction at the inflection point (*v_ip_*, see Figure 4) is a robust parameter that can be directly compared between different experiments and can be related to the levels of cellular biothiols. Representative examples of these experiments are shown in Figure 5. In particular, the fluorogenic biothiol reaction in a control cell in the absence of aggregates (Figure 5a) and in a cell with the addition of Aβ-647 aggregates that was incubated for 24 and 48 h (Figure 5b,c, respectively). These figures show how the probe diffuses across membranes into the cytosol and generates fluorescence when reacting with biothiols, undergoing a clear increase in the fluorescence emission as time passes. In the experiments with added Aβ-647 aggregates, we can clearly visualize how the aggregates surround the cell membrane in just a few minutes. As time proceeds, the size of these interacting aggregates considerably increased, which is consistent with the results described in the previous section. More repetitions for aggregates incubated during different times have been carried out, and representative examples are included in the Supplementary Materials (Figure S5).

**Figure 6:**
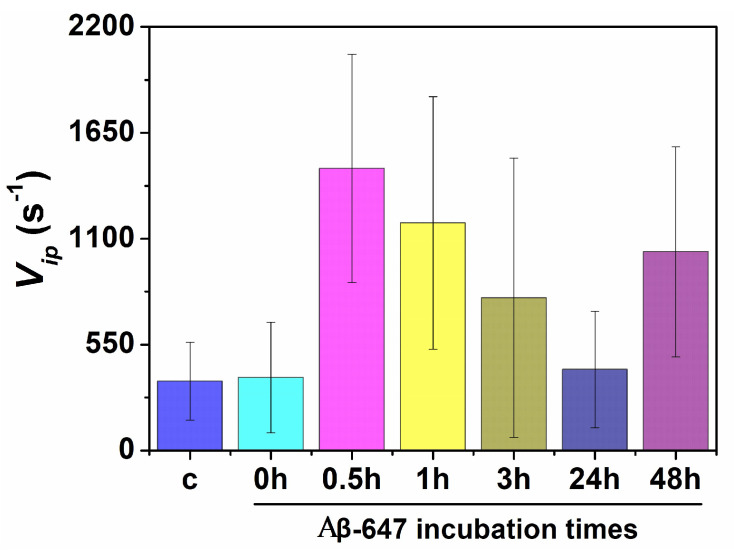
Biothiols sensing in cells interacting with Aβ-647 aggregates formed at different incubation times. The bar chart represents the average rate of the fluorogenic biothiol sensing reaction at the inflection point (*v_ip_*) for the controls (labeled as c) and for the cells interacting Aβ-647 aggregates formed at different incubation times (0, 0.5, 1, 3, 24, and 48 h of incubation). Error bars represent standard error of the mean (s.e.m.).

As mentioned above, between three and seven repetitions for all the measurements were performed using different cell cultures and different aggregate preparations to ensure the reproducibility of the results. Figure 6 shows the average values of the *v_ip_* rate as a measure of the biothiol levels upon the interaction of N2a cells with Aβ-647 aggregates and the corresponding controls. Consistently, a greater rate than the corresponding controls was found when the aggregates incubated for 0.5, 1, 3, and 48 h were added. Interestingly, nonincubated aggregates (0 h) did not cause an increase in the rate of the fluorogenic biothiol reaction, even though the interaction with the cell membrane was detected (Figure S5). These results support higher levels of biothiols in cells interacting with Aβ-647 aggregates formed after 30 min of incubation, which may be correlated with enhanced cellular stress and, hence, the notion that most cytotoxic species are formed during the early stages of aggregation.

To further understand the effect that the amyloid peptide can exert on neuronal cells, we also performed another assay following a different scheme, in which N2a cells were interacting with Aβ-647 aggregates for 20 min and then generated biothiols were detected by the fluorogenic probe. This experiment shows that during the time that the peptide aggregates were interacting with cells, a considerable accumulation of the peptide were formed at certain points of the membrane in accordance with the other results seen in this study. These results can be seen in the Supplementary Materials (Figure S6).

**Figure 7:**
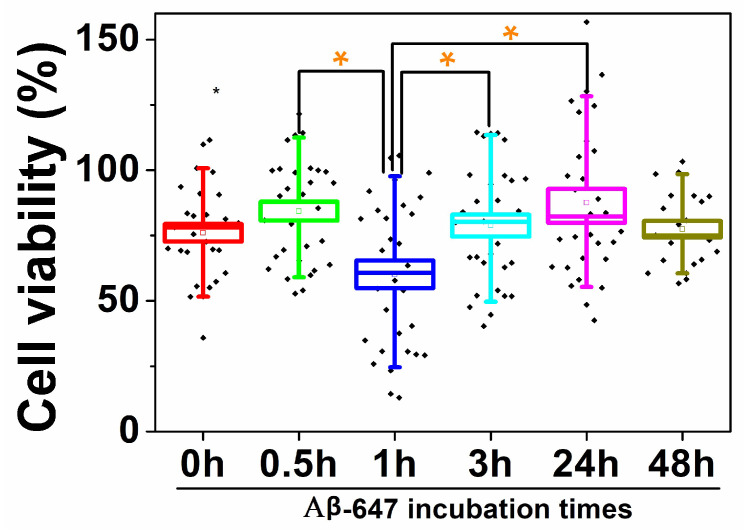
Box and whisker plot representing N2a cell viability upon the addition of Aβ-647 aggregates incubated for different times, expressed as the percentage of cell viability when compared with that in the control cells in the absence of aggregates. Boxes represent the standard error of the mean, and whiskers represent 90% of the results. Orange asterisks indicate populations that are significantly different from each other, with a 95% confidence interval.

To correlate the aforementioned results with actual cytotoxicity caused by Aβ-647 aggregates to N2a cells, we carried out cell viability assays using the commercial Cell Titer-Blue reagent (see Section 4 for details). We added aliquots of Aβ-647 aggregates incubated for different times (0, 0.5, 1, 3, 24, and 48 h) to cultured N2a cells, and let them interact for 76 h. Then, the Cell Titer-Blue reagent was added, and the fluorescence emission was compared between the experimental and control cells (with and without Aβ-647 aggregates added, respectively). In all cases, the addition of Aβ-647 caused a decrease in cell viability, between 11.6 and 41.3% (Figure 7). Importantly, we performed a statistical study for significant differences in toxicity using the Holm–Bonferroni test and found that the viability of the cells interacting with aggregates incubated for 1 h was significantly lower than that of the cells interacting with aggregates incubated for 0.5, 3, and 24 h, with a 95% confidence. These results suggest that Aβ-647 aggregates are moderately toxic to N2a cells. These values are in good agreement with previous toxicity studies of the Aβ peptide interacting with N2a cells, which reported viability values of almost 50% at a much higher peptide concentration (10 μM) than that used in our assay [42]. Regarding toxicity with other cell lines, it has also been shown Aβ42 aggregates at 10 μM that were previously incubated for 24 h at 37 °C, provoked a reduction in the cell viability of SH-SY5Y neuroblastoma cells by almost 20% [40], but the effect was even larger in primary neurons from E16 rat embryos in which Aβ42 aggregates at 20 μM caused a 65% reduction in cell viability [43].

**Figure 8:**
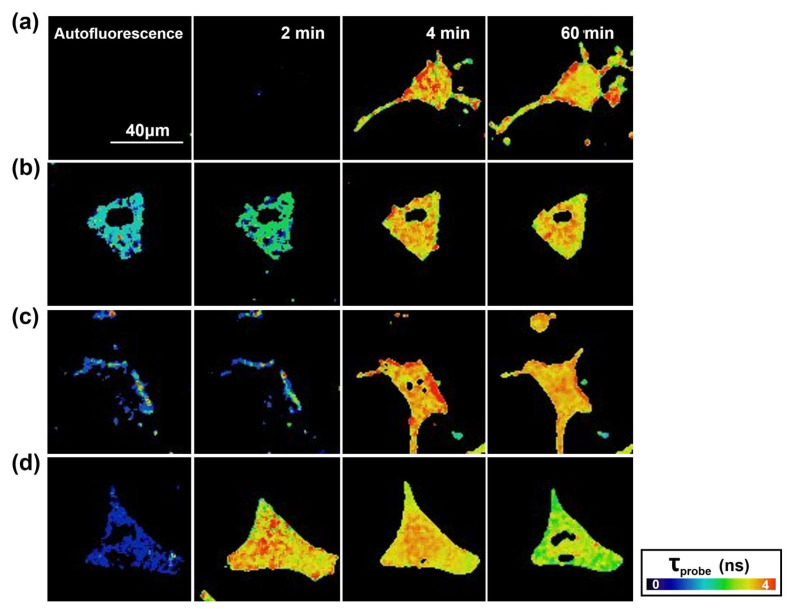
FLIM images of the released fluorophore after the fluorogenic reaction of GG-DNBS with biothiols in N2a cells without (**a**) and with the addition of Aβ42 aggregates, formed after incubating for 24 h (**b**) and 48 h (**c**,**d**). The pseudocolor scale represents the fluorescence lifetime of the probe, τ_probe_, between 0 and 4 ns.

In order to obtain additional information regarding the induction effect of different formed Aβ-647 aggregates on N2a neuronal cells, we took advantage of the fact that GG-DNBS is a dual sensor that is capable of not only simultaneously measuring levels of biothiols generated inside the cell but also detecting changes in the global levels of phosphate anions through the analysis of the fluorescence lifetime of the released fluorophore from the fluorogenic reaction, τ_probe_ [41,44]. The released fluorescent fragment undergoes changes in its fluorescence lifetime, influenced by the presence of phosphate anions, which promote a proton transfer reaction in the excited state of the fluorophore. Hence, we carried out an analysis of the FLIM images obtained in the previous experiments to determine appreciable changes in the intracellular phosphate levels upon interaction with Aβ-647 aggregates. Figure 8 shows FLIM images of the probe over time within the control cells (Figure 8a) and the cells treated with Aβ-647 peptide aggregates and incubated for 24 h (Figure 8b,c) and 48 h (Figure 8d), thus providing a global view of the phosphate levels present inside the cell. In the latter case, we can see that changes in the lifetime of the probe occur towards the end of the study period, decreasing its value. This finding suggests an increase in the global concentrations of phosphate ions caused by the incorporation of phosphate-enriched extracellular medium through permeabilized membranes.

## 3. Discussion

In this work, we report powerful microscopy tools to understand the interaction of Aβ42 aggregates with N2a neuronal cells at the single-cell level. The direct interaction of Aβ42 oligomers with the cell membrane, fostering cellular stress, lysis and death, was probed with a variety of methods, with a particular focus on dual-color PIE-FLIM due to its capability to provide multidimensional, rich images.

We first focused on the intra-oligomer FRET as a mean to identify different types of aggregates [30] and directly imaged their interaction with N2a cells using PIE-FLIM. For these experiments, we employed an equimolar mixture of labeled peptides, Aβ-488 and Aβ-647, incubated for 15 h. After this incubation period, it is expected that a large number of heterogeneous oligomers with different aggregation capacities and toxicities and nontoxic monomers coexist in equilibrium. Our PIE-FLIM results provide evidence of several key factors in the interaction of Aβ42 amyloid aggregates with N2a cells: (i) Aβ42 aggregates interact with the cellular membrane in just a few minutes. (ii) The interaction sites can act as effective seeds for the continuous recruitment of more aggregates. Hence, in-membrane aggregate growth is more likely to occur than a new interaction with a different cell. (iii) The former behavior results in some cells interacting with Aβ42 aggregates and others remaining intact. (iv) This interaction results in cell lysis after some period of interaction as well as aggregate seeding and growth, which involves certain conformational changes, as demonstrated by the appearance of the high-FRET aggregate population. These results provide a clear and unprecedented depiction of the cellular effect of amyloid aggregates, especially demonstrating that seeding takes place within the cell membrane. This is an important conclusion, which was consistently supported by repeated experiments, and that explains how Aβ42 aggregates can be toxic to cells, even at low nanomolar concentrations, since the local concentration within the cell membrane is cooperatively increased.

These results can be rationalized by previous studies that show a similar behavior for Aβ42 aggregates on the neuronal tissue of transgenic models of Drosophila melanogaster. Cellular accumulations of Aβ42 peptide in the form of diffuse nonamyloid plaques are correlated to neurodegeneration and premature death of the animal [45,46]. Likewise, in another work, in which a transgenic mouse model that overexpresses the human mutated amyloid precursor protein was used, clear neurodegeneration caused by the peptide was observed. Interestingly, neurodegenerative activity was found to be related to intraneuronal deposits of the peptide but not to the extracellular accumulation of Aβ42 [47]. The fact that a heterogeneous interaction of Aβ42 aggregates with the membranes of some neuronal cells was observed in our results suggests that this interaction occurs selectively—only in places with a high affinity for the peptide, such as certain membrane lipids [48]. This affinity can alter the properties of the membrane and interfere with its fluidity, promoting fibrillogenesis [49]. It is also known that the formation of amyloid structures occurs in lipid rafts containing a ganglioside cluster [50]. On the other hand, the evident ability of Aβ42 to induce the permeability of some cell membranes in this work can be related to the propensity of the peptide to form Ca^2+^-permeable channels [50]. It has been reported that some protofibers formed by Aβ behave as pore-forming structures that can alter cell activity and cause cell death [51]. Our results also agree with previous reports that describe the cellular uptake of Aβ42 aggregates, which occurs with the prerequisite of the rapid binding of β-sheet-rich aggregates to the cell membrane [52]. Therefore, our results are consistent with the idea that the entry of aggregates into the cell interior could be a crucial step in its cytotoxicity. Thus, our work provides significant information regarding the process of interaction, permeabilization and cell lysis that Aβ42 aggregates exert on cells. As membrane permeability induced by Aβ42 aggregates is observable only in certain cases here, we suggest that there are different types of aggregates that generate different levels of cellular toxicity, in line with other studies that show how certain aggregates of Aβ42 induce greater cellular permeability [8]. Among these different aggregates, those that are more stable and that compact than the initial aggregates could be the most harmful to cells [13].

Importantly, a common limitation of previous biophysical studies in terms of their physiological significance is that the formation of Aβ peptide amyloid fibrils was performed at high in vitro concentrations, whereas physiologically relevant Aβ concentrations lie in the low nanomolar range. Very recently, Lyubchenko and colleagues studied the interaction of Aβ42 peptides with membrane bilayers using AFM and computational experiments, concluding that aggregation at low concentrations of the peptide is triggered by interaction with the membrane [53]. Our results clearly support this model; we added nanomolar concentrations of incubated aggregates to the cell buffer, and we clearly detected the interaction with the membrane and subsequent seeding and growth.

In the second set of experiments, we focused on the cellular stress caused by Aβ42 aggregates using a fluorogenic sensor for biothiols and a powerful dual-channel microscopy to narrow our study to the single-cell level. The production of biothiols is associated with a complex metabolic response elicited by cellular stress that depends biologically on each individual cell and on how these individual cells respond to an adverse factor. Importantly, the dual-channel microscopy method allows us to directly visualize how Aβ-647 aggregates interact with N2a cells while simultaneously measuring biothiol levels. This is important because it allows for the direct elucidation of whether aggregates are interacting with the studied cell, thereby providing a deeper understanding on the actual effect of this interaction. We also detailed our investigation into the search for differences in the effect caused by aggregates formed in different stages of the aggregation process. Our results show that the interaction of aggregates with the cellular membrane plays a role in the enhanced cellular stress leading to toxicity. We found that aggregates formed in the initial 0.5–1 h of incubation cause the most cellular stress (Figure 6), which correlates well with our cell viability and toxicity experiments (Figure 7) as well as with previous toxicity studies [40,42,43] suggesting that aggregates formed at early stages of the aggregation process are the most powerful for inducing membrane permeability [8]. However, importantly, during the very early stages, the large majority of aggregates are nonfibrillar, but a dynamic population usually returns to monomers [54]. This oligomer kinetics reinforce the idea of a dynamic and critical seeding process within the cell membrane to foster cellular stress and cell lysis. The key link between Aβ42 toxicity and cellular stress is still obscure. However, Butterfield and colleagues suggested a close relationship with lipid peroxidation that takes place in the cell membranes of neurons promoted by the peptide [55], which may support the catalytic role of aggregates upon binding to the cell membrane.

**Figure 9:**
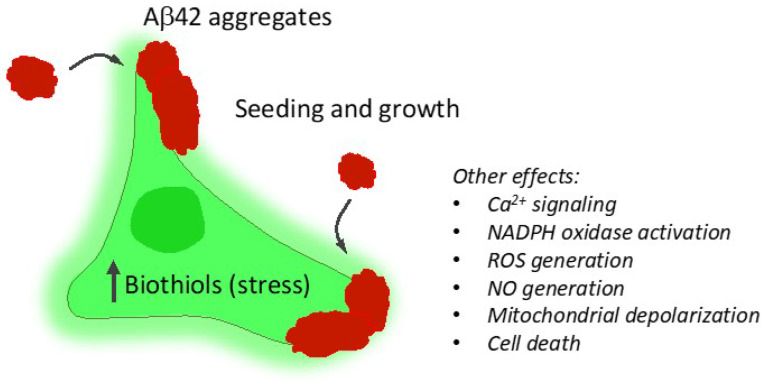
Main findings in this work. Aβ42 aggregates are bound to the cell membrane, acting as seeds for further growth. This membrane interaction is accompanied by increased cellular oxidative stress, finally leading to lysis and cell death.

Taken together, our results offer a broad picture of the relationship between Aβ aggregates interacting with neuronal cells, cellular stress, and neuronal toxicity (Figure 9). Amyloid aggregates formed in the early stages are the most harmful to N2a cells, which occur via interaction between the membrane and aggregates of a suitable size and structure, followed by subsequent aggregate seeding and growth, resulting in induced permeability until cellular lysis occurs in some cases. In summary, the tools that we presented herein are fully validated and have general applicability to further explore the cellular mechanisms underlying neurodegenerative diseases.

## 4. Materials and Methods

### 4.1. Materials

Lyophilized Aβ42 peptides labeled with HiLyte 488 dye (Aβ-488) and HiLyte 647 dye (Aβ-647), were obtained from Anaspec Peptide (Seraing, Belgium). A total of 0.1 mg of each peptide was dissolved in NH_3_ (1%) at a total concentration of 66 µM, sonicated in ice in an ultrasound bath for 30 min, distributed in aliquots and frozen immediately in liquid nitrogen to avoid aggregation. SSPE buffer (150 mM NaCl, 10 mM phosphate, and 1 mM EDTA) was acquired from Sigma-Aldrich (Madrid, Spain). For neuronal cells experiments, and Krebs buffer (130 mM NaCl, 2.5 mM KCl, 25 mM NaHCO_3_, 1.2 mM NaH_2_PO_4_, 1.2 mM MgCl_2_, and 2.5 mM CaCl_2_, pH 7.2) was freshly prepared. To adjust the pH of the buffers, NaOH and HCl (both from Sigma-Aldrich) were used. All chemical compounds were used without any further purification. Cell viability assays were carried out using a CellTiter-Blue™ viability assay (Promega Biotech, Madrid, Spain).

The N2a (ATCC^®^ CCL-131™) cell line used in these experiments is a cell line that comes from Mus musculus brain neuroblasts and presents neuronal stem cell and ameboid morphology. N2a cells were grown at 37 °C in Dulbecco’s modified Eagle’s medium (DMEM) from Sigma-Aldrich supplemented with 10% fetal bovine serum (FBS), 2 mM glutamine and 100 U/mL penicillin and 0.1 mg/mL streptomycin.

For the preparation of the biothiol probe, appropriate amounts of the powdered GG-DNBS (2,4-dinitrobenzenesulfinate derivative of 9-[1-(4-tert-butyl-2-methoxyphenyl)]-6-hydroxy-3H-xanthen-3-one) were dissolved in dimethyl sulfoxide (Sigma-Aldrich) to prepare a 0.36 mM stock solution. For imaging experiments, the fluorogenic probe was diluted in the buffer containing the cells, down to a final concentration of 0.25 μM.

### 4.2. Instruments

Dual-color PIE-FLIM experiments were performed on a MicroTime 200 system (PicoQuant GmbH, Berlin, Germany), as previously described [30]. As excitation sources, we used two pulsed laser diode heads (PicoQuant) at λ_ex_ = 470 and 635 nm, with a repetition rate of 20 MHz, and alternating in the nanosecond time regime for achieving PIE excitation. The excitation light was focused into the sample throughout a 100×, 1.4 numerical aperture (NA) oil immersion objective, which collected the fluorescence emission and focused it to the 75-μm confocal aperture. Fluorescence was then separated into two detection channels through a 600DCXR dichroic mirror, focusing the light on two single photon avalanche detectors (Perkin Elmer, Hopkinton, MA, USA). A 520/35 or 685/70 bandpass filter was employed to define the blue or red channel, respectively. A TimeHarp 200 time-correlated single-photon counting (TCSPC) module (PicoQuant, Berlin, Germany) was used for photon counting, data acquisition and imaging reconstruction.

We obtained raw images at a resolution of 0.26 µm/pixel and a temporal resolution of 232 ps/channel at the micro-time scale. Analysis of the images (separation and reconstruction of the different images and FLIM imaging) was performed in the SymPhoTime 32 software (PicoQuant). For FLIM imaging of the donor fluorophore, we fit the data to a single exponential decay function in the fluorescence decay traces obtained in each pixel after a 5 × 5 spatial binning using the maximum likelihood estimator (MLE) for parameter optimization.

We used in-house coded scripts in FiJi (distribution of ImageJ) [56] for the selection of colocalized pixels in the three images (*I_D_*, *I_FRET_*, and *I_A_* images) in the intra-oligomeric FRET experiments to selectively pick pixels corresponding to aggregates and scripts to quantify the average intensity per pixel in the regions of interest of the fluorogenic biothiol probe.

For the cell viability assays, an FP-8500 spectrofluorometer (Jasco), equipped with a microplate reader, was used to record the fluorescence emission spectra (λ_ex_ = 550 nm) of the CellTiter-Blue reagent in each well.

TEM images were obtained using a Libra 120 Plus TEM microscope (Carl Zeiss SMT, Oberkochen, Germany). It was operated at 120 kV and equipped with a LaB6 filament and an SSCCD 2 k × 2 k direct coupling camera. TEM images of samples incubated at different times were collected by adding aliquots on Formvar 300-mesh copper grids, washed twice with Milli-Q water and stained with uranyl acetate 1% (*w/v*).

DLS measurements were carried out on a Malvern Zetasizer µV, equipped with an 850 nm laser to avoid fluorescence interference with the 647-labeled peptides. DLS traces of the aggregates of Aβ-647 and Aβ-488 formed in NH_3_, pH 12, and in SSPE buffer, pH 7.4, after 20 min of incubation were collected using a 2 μL quartz cuvette.

### 4.3. Aβ Amyloid Aggregation

Aβ42 aggregates containing equimolar amounts of Aβ-488 and Aβ-647 used in the FRET assay were prepared at a total concentration of 0.5 µM for each peptide in SSPE buffer, and incubated for 15 h at a physiological temperature (37 °C) with continuous agitation (360 rpm). Aβ-647 aggregates, used in the experiments with the fluorogenic biothiol probe, were described as above at a total concentration of 1 µM. Aliquots were collected at different incubation times (0, 0.5, 1, 3, 24, and 48 h) and immediate frozen in liquid nitrogen until use.

### 4.4. Cell Viability Assays

CellTiter-Blue^®^ (Promega) viability assay, based on the resazurin → resorufin fluorogenic reaction, was performed to study the cytotoxicity of Aβ42 aggregates. Cell viability of the samples treated with Aβ42 aggregates was evaluated by the comparison with untreated controls, for which a cell viability of 100% is assumed. Cells were grown, up to a population density of 10^3^ cells/well, in black 96-well plates with 100 µL of DMEM plus 10% FBS per well. The medium was removed after 24 h of cell culture at 37 °C. Then, 100 µL aliquots of Aβ-647 incubated for different times were added to cells for 76 h. Finally, 20 µL (20% *v/v*) of CellTiter-Blue reagent was added to each well and incubated for 20 min at 37 °C before fluorescence emission was measured.

## Supplementary Materials

The following are available online at https://www.mdpi.com/1422-0067/21/14/5035/s1. Figures S1–S4. Supplementary figures of the interaction of equimolar mixtures of labeled Aβ-488 and Aβ-647 peptides (incubated for 15 h) with N2a cells. Figure S5. Additional examples of cell stress assays with the biothiol probe (green channel), upon the addition of previously incubated Aβ-647 aggregates (red channel). Figure S6. Second strategy for the assay of cellular stress induced by Aβ-647 aggregates.

## Abbreviations

FLIM	Fluorescence lifetime imaging microscopy
FRET	Fluorescence resonance energy transfer
PIE	Pulsed interleaved excitation
Aβ42	β-Amyloid (1-42) peptide
Aβ-488	β-Amyloid (1-42) peptide labeled with HiLyte Fluor 488
Aβ-647	β-Amyloid (1-42) peptide labeled with HiLyte Fluor 647
GG-DNBS	Granada Green dinitrobenzene sulfonate
TEM	Transmission electron microscopy
DLS	Dynamic light scattering
TCSPC	Time-correlated single-photon counting
