# Supplementary material for: Seeding and Growth of β-Amyloid Aggregates upon Interaction with Neuronal Cell Membranes
Source: Int J Mol Sci. 2020 Jul 16;21(14):5035. doi: 10.3390/ijms21145035 (PMC7404110; doi:10.3390/ijms21145035)
Supplement: Supplementary file 1 [file ijms-21-05035-s001.pdf]

Supplementary Material

S1. Supplementary Figures of the Interaction of Equimolar Mixtures of Labeled Aβ-488 and Aβ-647 Peptides (Incubated for 15 h) with N2a Cells

Figures S1–S4 show four representative examples of the time course of the interaction of mixed Aβ-488 and Aβ-647 aggregates with N2a cells. The fluorescence intensity images in the three detection windows (I_D, I_{FRET}, and I_A) and FLIM images of the donor fluorophore, τ_D, are shown in parallel, with arrows indicating different detected cells for identification purposes. Histograms in the rightmost column represent τ_D distributions for only pixels selected as aggregates. The distributions were fitted to one or two (if needed) Gaussian functions (lines).

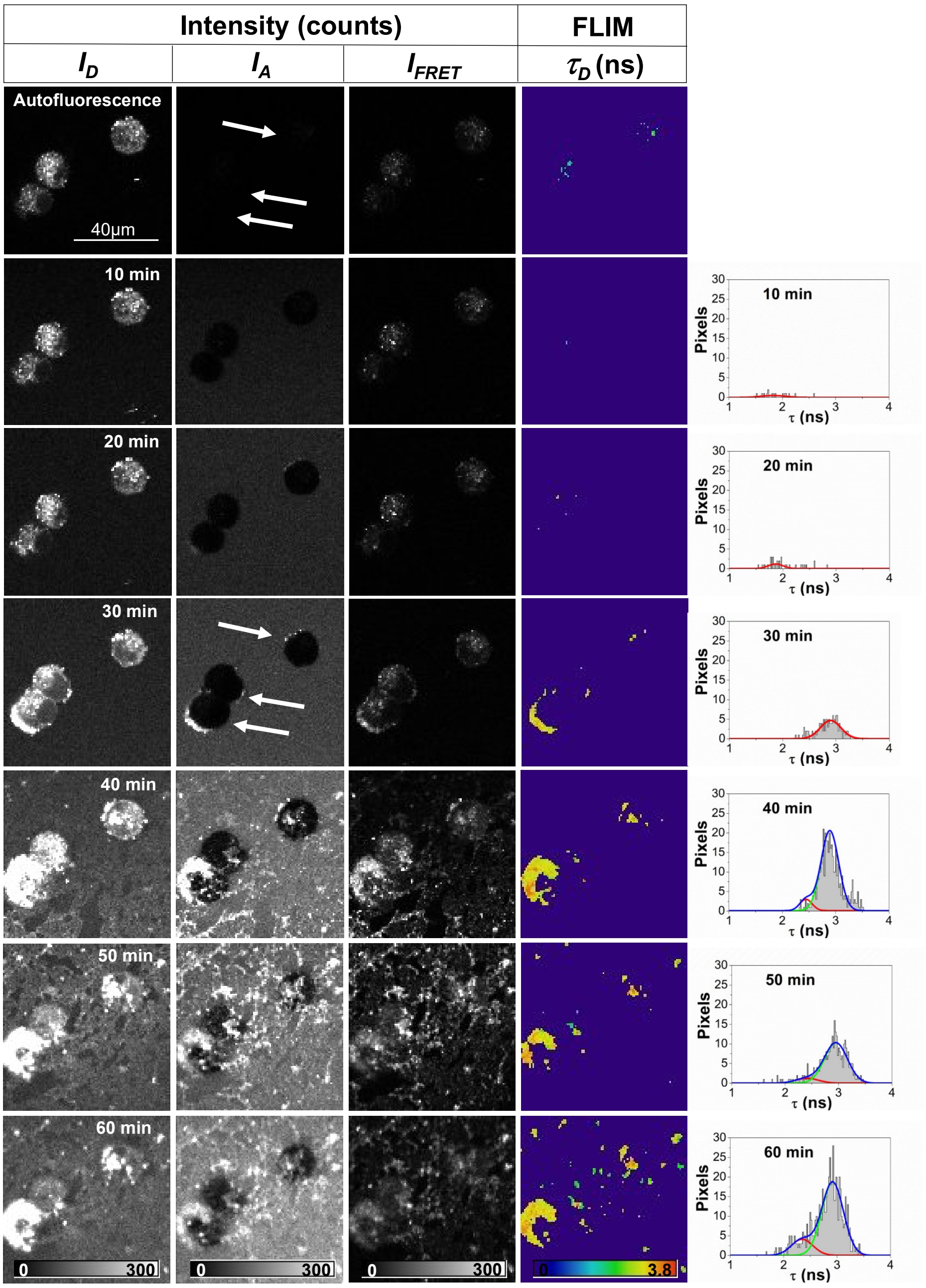

Figure 1. Time course of the interaction of labeled Aβ42 aggregates with N2a cells. Representative example 1.

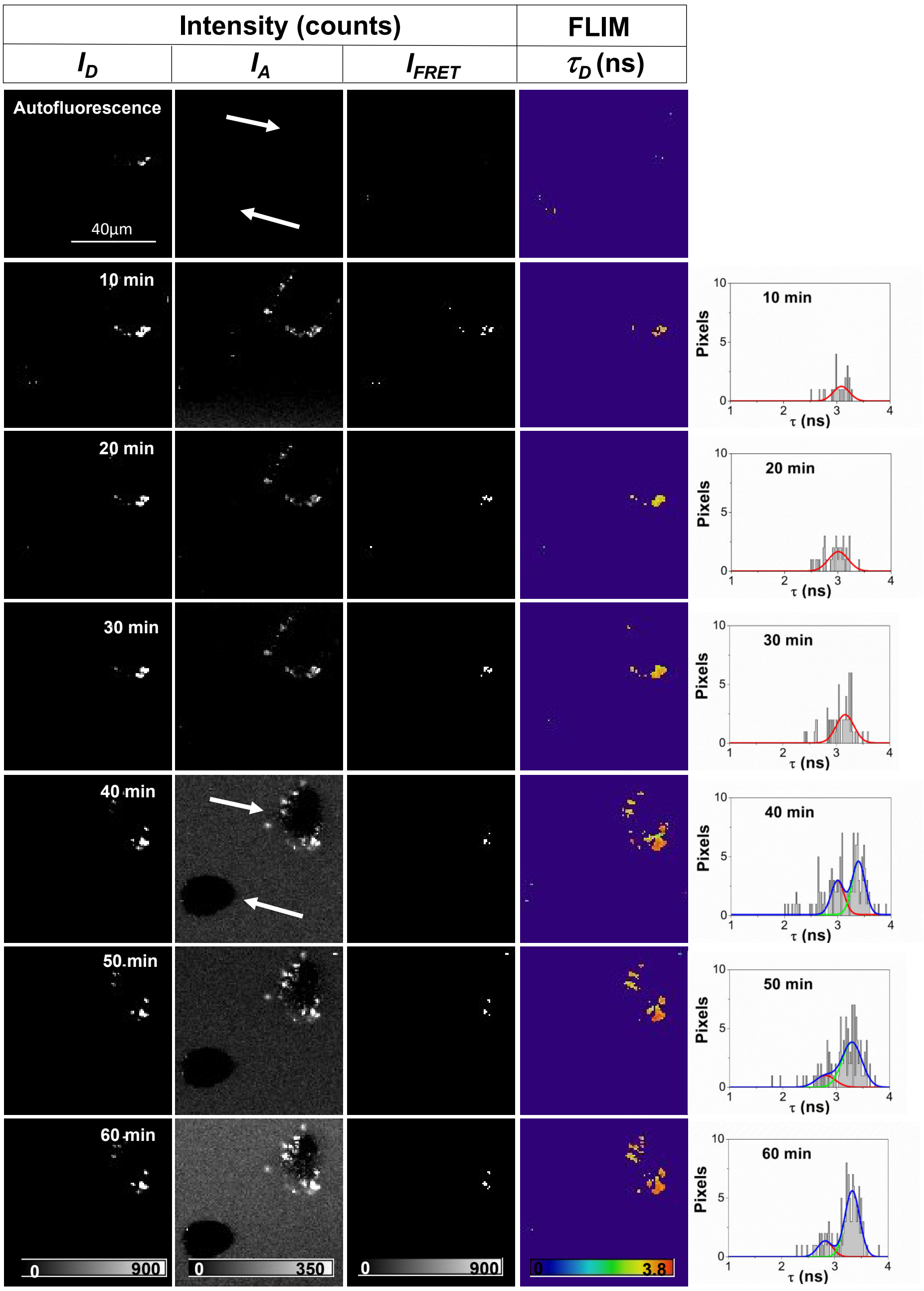

Figure 2. Time course of the interaction of labeled Aβ42 aggregates with N2a cells. Representative example 2.

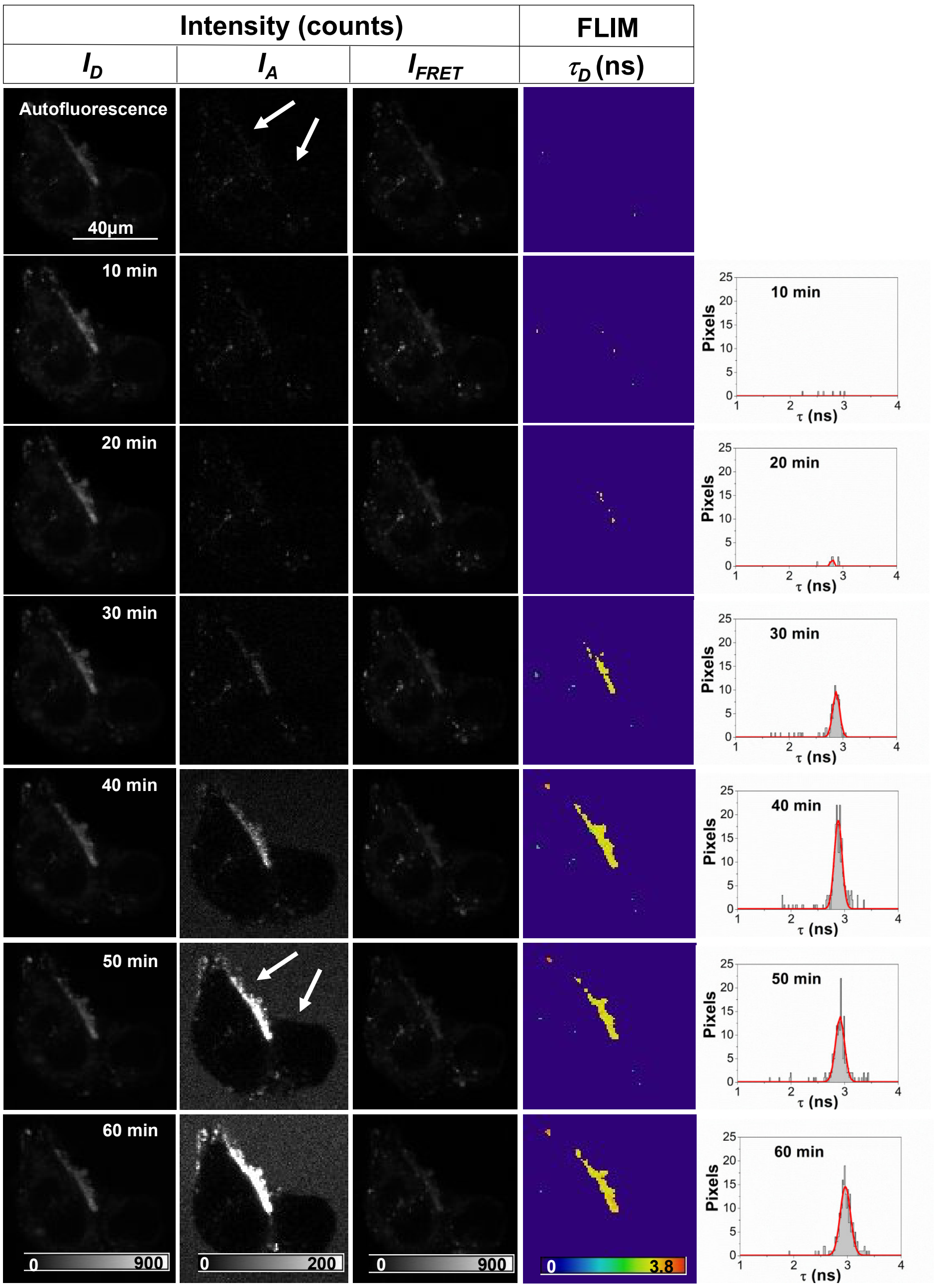

Figure 3. Time course of the interaction of labeled Aβ42 aggregates with N2a cells. Representative example 3.

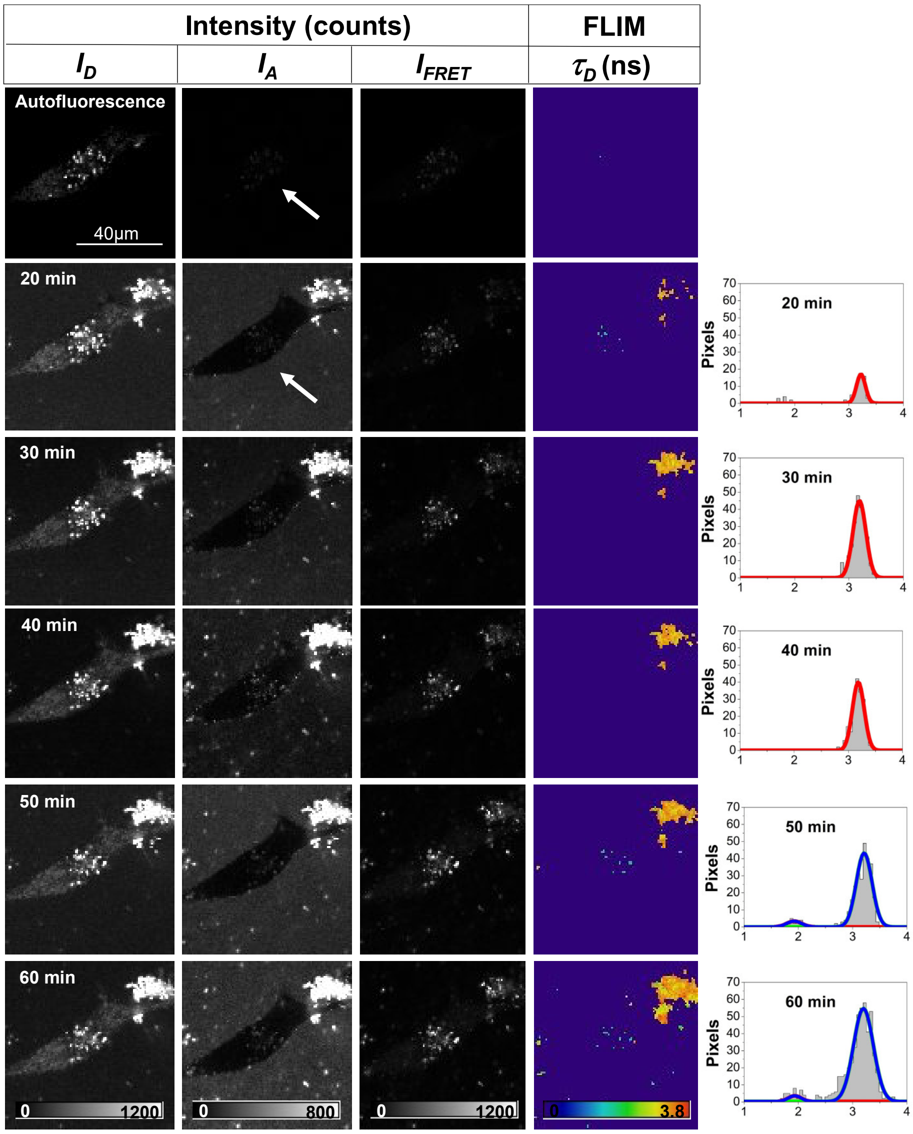

Figure 4. Time course of the interaction of labeled Aβ42 aggregates with N2a cells. Representative example 4.

S2. Additional Examples of Cell Stress Assays With the Biothiol Probe

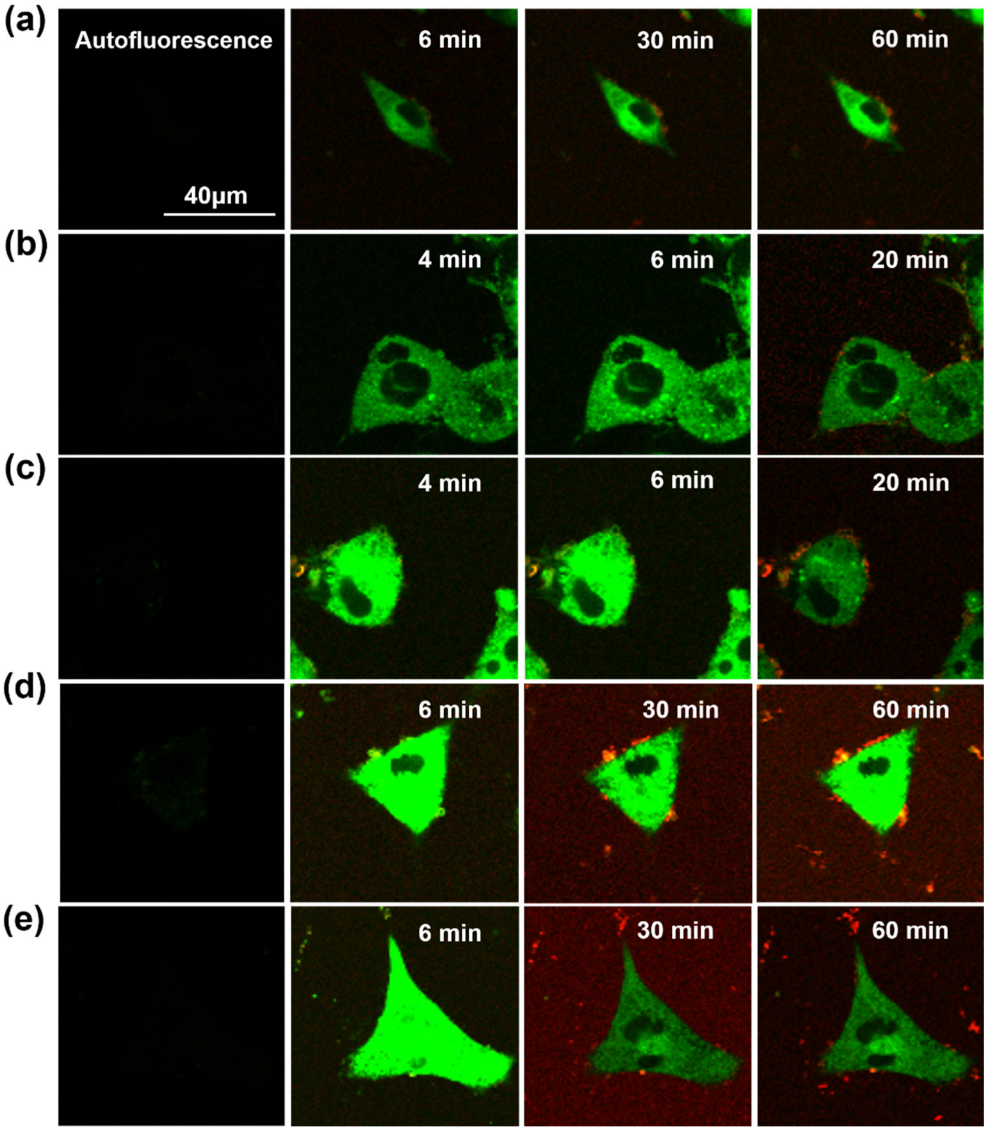

Figure 5. Additional examples of cell stress assays with the biothiol probe (green channel), upon the addition of previously incubated Aβ-647 aggregates (red channel).The images are representative examples of the effect of Aβ-647 aggregates incubated for 0 (**a**), 1 (**b**), 3 (**c**), 24 (**d**) and 48 h (**e**).

S3. Second Strategy for the Assay of Cellular Stress Induced by Aβ-647 Aggregates

In this strategy, N2a cells and Aβ-647 aggregates (red channel) interacted for 20 min prior to the addition of the biothiol fluorogenic probe (green channel), GG-DNBS. Once the fluorogenic probe is added, dual-color image collection starts. Times depicted in Figure S6 are counted from the addition of the GG-DNBS probe.

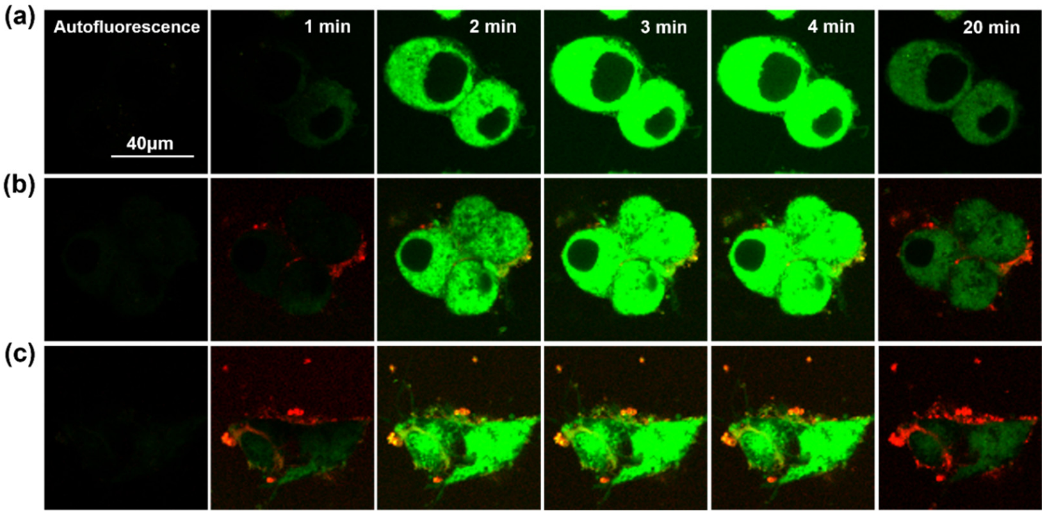

Figure 6. Second strategy for the assay of cellular stress induced by Aβ-647 aggregates. (**a**) Control experiment without added aggregates; b and c) addition of Aβ-647 aggregates incubated during 0 (**b**) and 1 h (**c**).
